# Bisexual Individuals Envisioning Aging Futures: Emergent Outlooks on Later Life

**DOI:** 10.1093/geroni/igaf122.2399

**Published:** 2025-12-31

**Authors:** Zhiqi Yi, Olivia Lafountain, Xavier Noriega, Austin Oswald, Sarah Jen

**Affiliations:** University of Kansas, Lawrence, Kansas, United States; University of Kansas, Lawrence, Kansas, United States; University of Kansas, Lawrence, Kansas, United States; Dalhousie University, Halifax, Nova Scotia, Canada; University of Kansas, Lawrence, Kansas, United States

## Abstract

The outlooks individuals envision their future shape their expectations and approach to later life. While bisexual older adults report distinct life experiences from lesbian, gay, and heterosexual counterparts, their outlook on later life remains unclear. Guided by the queer aging futures perspective (Sandberg & Marshall, 2017), this study examines future outlooks among 21 bisexual-identified adults in the U.S. (aged 51–71, Mean=58.6) through semi-structured life review interviews analyzed via inductive thematic coding. Four future outlooks emerged, forming two contrasting but not mutually exclusive pairs: Exploratory vs. Settling-Down and Affirmation vs. Fraught. Individuals with exploratory outlooks anticipated continued fluidity in their own sexuality, gender, and social connections, embracing the potential of rich and flexible social networks and communities in the future. Individuals with settling-down outlooks desire to settle into a comfortable and consistent day-to-day experience with their current families and loved ones. Affirmation-oriented individuals expressed optimism, envisioning futures of acceptance, belonging, and LGBTQ+ activism and aging-supportive communities. Conversely, fraught outlooks entailed concerns about aging, end-of-life challenges, and potential setbacks in LGBTQ+ rights. Consistent with the life course perspective, these outlooks reflect earlier life experiences that may shape individuals’ anticipated futures. We see the exploratory outlook as reflecting particular strengths of bisexual communities, such as a capacity for openness, fluidity, and flexibility that could inspire adaptability in anticipated later lives, not just for bisexual aging but for aging populations broadly. These findings illustrate possible multiplicities and complexities of queering aging futures with distinctly bisexual interpretations of what later life might entail.

